# Comparison of survival and post-operation outcomes for minimally invasive versus open hepatectomy in hepatocellular carcinoma: A systematic review and meta-analysis of case-matched studies

**DOI:** 10.3389/fonc.2022.1021804

**Published:** 2022-10-20

**Authors:** Bing Fu, Jin-Rui Zhang, Pin-Sheng Han, Ya-Min Zhang

**Affiliations:** ^1^ The First Central Clinical School, Tianjin Medical University, Tianjin, China; ^2^ Department of Hepatobiliary Surgery, Tianjin First Central Hospital, Tianjin, China

**Keywords:** minimally invasive hepatectomy, open hepatectomy, hepatocellular carcinoma, meta-analysis, robotic hepatectomy

## Abstract

**Background:**

With the rapid development of minimally invasive techniques and instruments, more and more patients begin to accept minimally invasive surgery. Minimally invasive hepatectomy (MIH) has obvious advantages in terms of surgical incision, but there is still no strong evidence of its long-term survival effect.

**Purpose:**

The primary objective of this study was to compare long-term survival outcomes between MIH and Open hepatectomy (OH) in hepatocellular carcinoma based on high-quality case-control studies.

**Methods:**

The study on the comparison of MIH (including RH or LH) and OH in the treatment of HCC from the date of establishment to June 1, 2022 was searched through PubMed, Web of Science, Embase and Cochrane Library databases. The main results were long-term overall and disease-free survival and short-term postoperative effect; All studies were conducted according to PRISMA guidelines, and meta-analysis of random effect models was adopted.

**Results:**

43 articles included 6673 patients. In these studies, the data from 44 studies need to be extracted and pooled in the meta-analysis. Our results showed that compared with OH group, OS (HR 1.17; 95%CI 1.02, 1.35; P=0.02) and DFS (HR 1.15; 95%CI 1.05, 1.26; P=0.002) in MIH group were slightly lower than those in OH group. The operation time (Z=2.14, P=0.03, MD8.01, 95% CI: 2.60–13.42) was longer than OH group. In terms of length of hospital stay (Z=10.76, p<0.00001, MD -4.0, 95% CI: -4.72 to -3.27), intraoperative blood loss (Z=5.33, P<0.00001, MD -108.33, 95% CI: -148.15 to -68.50), blood transfusion rate (Z=5.06, p<0.00001, OR=0.64, 95% CI 0.54 to 0.76, I^2^ = 0%), postoperative complications (Z=9.24, p<0.00001, OR = 0.46, 95% CI 0.39 to 0.55, I^2^ = 21%), major morbidity (Z=6.11, p<0.00001, OR=0.46, 95% CI 0.39 to 0.59,I^2^ = 0%), R0 resection (Z=2.34, P=0.02, OR=1.46, 95% CI 1.06 to 2.0, I^2^ = 0%) and mortality(Z=2.71,P=0.007, OR=0.56, 95% CI 0.37 to 0.85), the MIH group was significantly better than the OH group. The meta-analysis showed no significant difference in terms of major hepatectomy Z=0.47, P=0.64, OR=1.04, 95% CI 0.89 to 1.22, I^2^ = 0%), anatomical resection (Z=0.48, P=0.63, OR=0.92, 95%CI 0.67 to 1.27), satellite nodules (Z=0.54, P=0.59, OR=0.92, 95%CI 0.69 to 1.23, I^2^ = 0%), microvascular invasion (Z=1.15, P=0.25, OR=1.11, 95%CI 0.93 to 1.34, I^2^ = 0%) and recurrence (Z=0.71, p=0.48, OR=0.94, 95% CI 0.78 to 1.12, I^2^ = 19%).

**Conclusion:**

This study is the first to compare the clinical efficacy of MIH and OH in the treatment of HCC based on a high-quality propensity score matching study. The results show that in terms of long-term survival outcomes (OS and DFS), although the gap between MIH and OH is not obvious, OH was better than MIH on the whole. However, in terms of short-term postoperative outcomes (post-operation outcomes), MIH was slightly better than OH.

**Systematic review registration:**

https://www.crd.york.ac.uk/PROSPERO/, identifier CRD42022332556.

## Introduction

According to global cancer statistics in 2020: primary liver cancer is the seventh most commonly diagnosed cancer and the third leading cause of cancer death worldwide, with approximately 906,000 new cases and 830,000 deaths (1). According to different periods of patients with hepatocellular carcinoma, the treatment methods are different. For early cancer, patients can choose surgical resection, liver transplantation or radiofrequency local ablation. But it is well known that surgery is the most effective treatment for hepatocellular carcinoma (2).

Nowadays, with the continuous development of surgical techniques and instruments, more and more people begin to appreciate the advantages of minimally invasive surgery, such as less trauma, less bleeding and faster recovery. In addition, the confidence and expectation of doctors and patients on postoperative efficacy have also increased. In 1991, a research team from the United States (Reich H and McGlynn F et al.) first reported two cases of laparoscopic hepatectomy for benign liver lesions, which opened the era of minimally invasive hepatectomy for liver tumors (3). Since then, with the mastery of laparoscopic hepatectomy technology, surgeons have gradually realized the great advantages of minimally invasive liver resection. In recent years, Da Vinci surgical system(DaVSS)has come to be known and widely used in the field of surgery. Inspired by this, in 2006, Ryska et al. reported for the first time two cases of robotic hepatectomy (RH). Both patients recovered well without any complications (4). This case report proves the technical feasibility of this minimally invasive method, which lays a solid foundation for the application of robot technology in the field of hepatectomy, and it also shows the further development of MIH technology. Although these studies have shown encouraging results, the strength of MIH is mainly reflected in short-term outcomes after surgery, and there is a lack of comparison of such long-term outcomes as overall survival and progression-free rates. The purpose of this study was to compare the high-quality Case-control study of MIH with OH for hepatocellular carcinoma (HCC), so as to determine whether MIH has obvious advantages in long-term and short-term curative effects.

## Materials and methods

### Search strategy

We searched PubMed, Web of Science, Embase, and Cochrane Library databases for studies comparing MIH (including RH or LH) and OH for HCC from inception to June 1, 2022. We also manually searched through relevant references to identify other relevant studies. The detailed search strings are shown in **Supplementary File 1**. Review was reported following the PRISMA guidelines. The protocol of this study was registered in the International Prospective Register of Systematic Reviews (PROSPERO), CRD42022332556.

### Inclusion/exclusion criteria

The following inclusion criteria were considered in this study (1): population: patients with resectable hepatocellular carcinoma; (2) Intervention and Comparison: methods of hepatectomy (RH or LH vs OH); (3) outcomes: overall survival, disease-free survival, overall morbidity, blood loss, conversion rate, operative time, R0 resection rate, length of hospital stay, blood transfusion rate, postoperative complication, major morbidity, anatomical resection, satellite nodules, microvascular invasion, major hepatectomy, and recurrence; (4) Study design: Case-Matched Studies (High-quality Propensity score matching research); Non-comparable study, case report, editorial, meta-analysis, review, studies published in languages other than English, small sample size (less than 20), no survival-related data, low quality studies were excluded. Duplicate data for the same institution, we selected the most comprehensive study.

### Study selection

We retrieved the titles and abstracts of individual studies using keywords search. Two investigators (HPS and ZJR) screened the literature based on prespecified inclusion and exclusion criteria. Any differences are settled by discussion and consensus. In the event of disagreement between the two investigators, a third investigators (ZYM) involved in the decision-making.

### Quality assessment

The quality of non-randomized studies was evaluated by using the Newcastle–Ottawa Scale (NOS). The standard included three categories—patient selection (four points), comparability of the study groups (two points), and ascertainment of exposure or outcome (three points). Studies with a cumulative score of ≥ 6 points were considered to be of high quality. Two investigators (HPS and ZJR) independently evaluated the quality of the selected articles, exchanged different opinions, and sought third-party quality evaluation results.

### Data extraction and outcome measures

Post-operation outcomes assessed were overall morbidity, blood loss, conversion rate, operative time, R0 resection rate, length of hospital stay, blood transfusion rate, postoperative complication, major morbidity, anatomical resection, satellite nodules, microvascular invasion, and major hepatectomy. Long-term survival outcomes evaluated were overall survival (OS) as well as DFS (disease-free survival) for HCC. Data on first author, publication year, type of study, number of patients enrolled, patients’ age and sex, tumor size and number, types of surgery, and liver function status (HBV and cirrhosis) were also extracted. To maintain data consistency across meta-analyses, we converted medians and their ranges to sample means and standard deviations (5). In addition, when the relevant hazard ratio (HR) was not directly available, data from Kaplan-Meier curves were considered (6).

### Statistical analysis

RevMan version 5.4 software (Cochrane Collaboration, Copenhagen, Denmark) was used for data analysis. The continuous variables were assessed by standardized difference (SD), dichotomous variables by odds ratio (OR) and 95% confidence interval (CI), and survival outcomes by HR. The mean and standard deviation (SD) were calculated from the median and interquartile ranges using the method proposed by Wan X et al (5). For those studies that did not have hazard ratio (HR) and standard errors or corresponding 95%CI, data in the Kaplan-Meier curves provided by published studies were evaluated using the approach suggested by Tierney et al (6). The fixed effect model was used to calculate all pooled results. Heterogeneity was assessed by calculating I^2^, and results greater than 50% were considered significant heterogeneity. In addition to, we also performed analyses of sensitivity and publication bias to explore heterogeneity between studies. P<0.05 was considered to indicate a statistically significant difference.

## Results

### Study selection and characteristics

**Figure 1:**
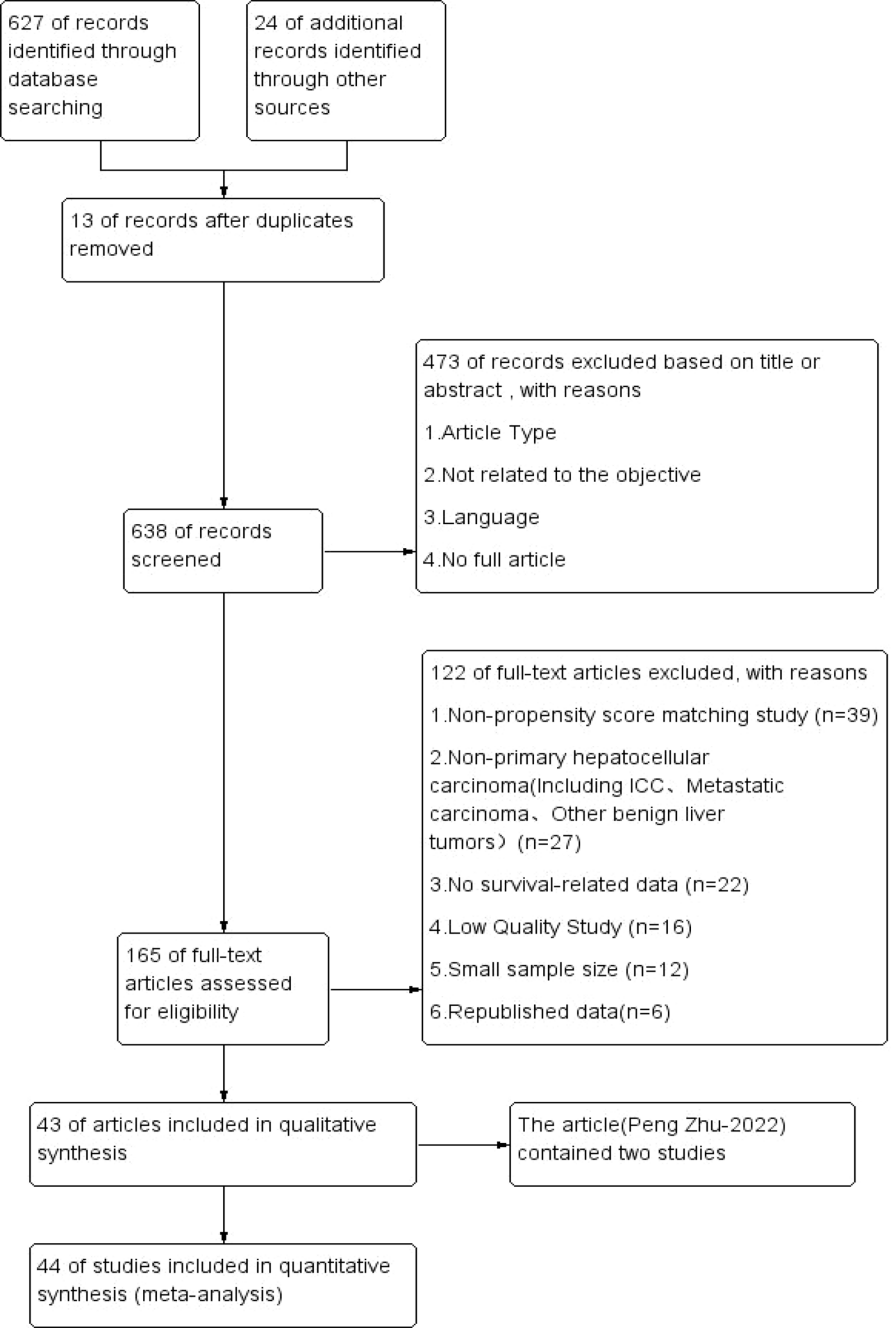
PRISMA flowchart of the established screening strategy.

We identified 627 articles from the database and searched 24 articles manually. After removing duplicates (13 studies), the total number of studies was 638. Of these, 473 were excluded by assessing titles or abstracts, and the remaining 165 studies underwent full-text evaluation. Ultimately, this meta-analysis included 44 studies summarized from 43 articles (Peng zhu-2022 contained two studies) (7). **Figure 1** displays a flowchart of the established screening strategy. The basic characteristics of the 44 studies included in this meta-analysis, including the author, publication year, tumor size and so on, are shown in **Table 1**. These studies were published between 2011 and 2022, with study periods ranging from 1990 to 2019. Among them, 42 studies (7–48) compared the LH group with the OH group, and the remaining 2 studies (7, 49) focused on the RD group with the OH group. The quality evaluation of individual studies is shown in

**Table 1:** All studies are considered to be of high quality.

Source		Approach(MIH/OH)	Number of patients(MIH/OH)	Sex(male)(MIH/OH)	Age(range)(MIH/OH)		Tumor size(range)(MIH/OH)		HBV(MIH/OH)		Single/multiple (MIH/OH)		Conversion	Cirrhosis (MIH/OH)		NOS quality score
AntonellaDelvecchio-2020	8	MIH/OH	38/84	29/61	75 (70–82)	74.3(70–86)	4.0(3.0–16.0)	7.0(1.5–14.0)	10	16	33(5)	68(16)	NA	23	37	8
AoxiaoHe-2021	9	MIH/OH	26/78	11/33	56.1 ± 10.6	52.0 ± 12.2	7.50 ± 3.51	7.55 ± 3.88	NA	NA	NA	NA	NA	16	45	6
Chen(2017)	49	RH/OH	81/81	61/65	60.6 (27-89)	60.0 (32-86)	3.2 (1.0-9.0)	3.3 (0.8-10.5)	NA	NA	NA	NA	NA	37	38	6
CheungTT-2013	10	MIH/OH	32/64	22/50	59.5 (39–79)	61 (29–82)	2.5 (1–10)	3 (1–10)	26	49	NA	NA	NA	32	64	7
ChongLAI-2016	11	MIH/OH	28/33	24/28	56.5 ± 12.6	52.8 ± 11.8	3.0 ± 1.1	3.3 ± 1.1	23	29	26 (2)	31 (2)	NA	18	22	8
DaiHoonHan-2015	12	MIH/OH	99/198	73/151	54.25 ± 11.07	54.70 ± 9.21	2.51 ± 1.14	2.58 ± 1.09	88	172	92 (7)	173 (25)	8 (8.1%)	53	122	9
Doo-HoLee-2019	13	MIH/OH	58/58	34/35	57.0 (33-82)	59.0 (34-81)	2.35 (0.7-14.0)	2.60 (1.1-14.5)	53	54	NA	NA	NA	35	39	7
FeiLiu-2019	14	MIH/OH	67/67	59/57	54.28 ± 12.03	53.06 ± 13.42	5.04 ± 2.68	5.17±2.22	54	55	57 (10)	58 (9)	2 (2.9%)	45	41	9
HadrienTranchart-2010	15	MIH/OH	42/42	15/14	63.7 ± 13.1	65.7 ± 7.1	35.8 ± 17.5	36.8 ± 20.9	NA	NA	NA	NA	2 (4.7%)	31	34	7
Ho-SeongHan-2015	16	MIH/OH	88/88	72/74	60 (26-81)	59.5 (20-85)	3 (1-12)	3 (1.5-15)	NA	NA	67 (21)	70 (18)	8 (9.1%)	55	52	8
JiangX-2016	17	MIH/OH	59/59	42/38	51 (36-68)	50 (38-70)	3 (2-5)	3 (1-6)	35	32	NA	NA	NA	NA	NA	6
JonghunJ.Lee-2015	18	MIH/OH	43/86	29/69	62.0 (30–86)	63.0 (34–84)	5.4 (2–16)	4.4 (2–14)	19	52	41 (2)	81 (4)	6 (14.0%)	18	33	9
JongManKim-2018	19	MIH/OH	37/37	30/31	58 (34–78)	58 (34–78)	2.8 (0.9–11.5)	2.8 (1.1–10)	27	31	NA	NA	1 (2.7%)	15	20	8
KeChen-2019	20	MIH/OH	38/38	31/32	56.0 ± 10.3	55.2 ± 11.1	7.3 ± 3.4	7.6 ± 4.2	35	33	32 (6)	32 (6)	7 (18.4%)	34	34	9
KeunSooAhn-2014	21	MIH/OH	51/51	36/40	58.2 ± 10.4	57.1 ± 10.6	2.6 ± 1.5	2.8 ± 1.2	40	37	51 (0)	51 (0)	5 (9.8%)	NA	NA	8
KimH-2013	22	MIH/OH	29/29	22/19	54.62 ± 9.16	53.90 ± 10.08	3.59 ± 2.17	4.28 ± 2.55	24	27	24 (5)	28 (1)	NA	18	19	8
Kit-ManHo-2021	23	MIH/OH	45/90	37/72	62 (57.5-68.0)	62 (54.75-71.00)	3.5 (2-5)	4 (3-5)	42	72	37 (8)	70 (20)	5 (11.1%)	26	58	9
KomatsuS-2016	24	MIH/OH	38/38	33/34	61.7 ± 16.1	61.5 ± 12.2	85.5 (20–180)	52.5 (23–130)	9	10	22 (16)	22 (16)	13 (34.2%)	NA	NA	8
L.Xiang-2016	25	MIH/OH	128/207	109/171	50.9 ± 11.9	50.5 ± 10.7	6.7 ± 1.5	6.9 ± 1.5	106	172	128 (0)	207 (0)	12 (9.4%)	104	167	9
LanyunLuo-2015	26	MIH/OH	53/53	38/35	49 (36-72)	51 (38-68)	3 (2-5)	3 (1-6)	41	38	NA	NA	NA	NA		6
LeeKF-2011	27	MIH/OH	33/50	24/40	59 (36–85)	58.5 (32–81)	2.5 (1.5–9)	2.9 (1.2–9)	22	46	31(2)	41 (9)	6 (18.2%)	28	32	9
Masateru Yamamoto-2019	28	MIH/OH	58/58	39/30	71 (34–89)	72 (45–88)	17 (2–42)	16 (8–50)	10	13	NA	NA	NA	NA	NA	6
MeguroM-2015	29	MIH/OH	35/35	23/27	70 (64–75)	66 (55–72)	2.5 (2.0–3.1)	3.0 (2.0–3.5)	15	14	28 (7)	29 (6)	NA	NA	NA	7
MemeoR-2014	30	MIH/OH	45/45	35/37	62 (34–75)	60 (43–80)	3.2 (0.9–11)	3.7 (0.1–15)	16	13	NA	NA	NA	45	45	7
Peng Zhu-2022	7	RH/OH	56/56	45/44	52 (28-72)	53 (21-73)	3.3 (1.0-12.5)	4.0 (1.2-14.0)	49	43	52 (4)	54 (2)	8 (14.3%)	NA	NA	8
Peng Zhu-2022	7	MIH/OH	56/56	47/44	53 (24-72)	54 (21-73)	3.3 (1.1-14.3)	4.0 (1.2-14.0)	48	43	53 (3)	54 (2)	7 (12.5%)	NA	NA	8
Sam-YoulYoon-2014	31	MIH/OH	58/174	45/130	54.3 (49–63)	55.0 (49–61)	2.87 (0.70–4.9)	3.04 (0.20–4.9)	54	165	58(0)	174 (0)	0	NA	NA	8
SpositoC-2016	32	MIH/OH	46/46	28/35	66 (40–85)	68 (49–83)	2.6 (1.0–6.5)	2.2 (1.0–8.5)	6	10	37 (9)	35 (11)	NA	46	46	8
StefanoDiSandro-2018	33	MIH/OH	75/75	42/51	68.6 (61.3,75.5)	67.1 (61.2,75)	2.5 (2,3)	2.5 (1.8,3.3)	7	11	67 (8)	65 (10)	NA	75	75	8
Sung-JinKim-2014	34	MIH/OH	70/76	58/58	59.30 ± 9.43	59.30 ± 9.43	2.58 ± 1.44	2.58 ± 1.44	46	54	NA	NA	6 (8.57%)	NA	NA	7
TakeshiTakahara-2015	35	MIH/OH	387/387	262/261	66.42 ± 9.84	66.42 ± 9.84	28.8 ± 15.1	28.8 ± 15.0	91	100	NA	NA	18 (4.56%)	322	310	8
TanakaS-2015	36	MIH/OH	20/20	17/14	70 (66-73)	71 (67-75)	2.3 (2.0–2.7)	2.3 (1.9–2.8)	4	2	18 (2)	17 (3)	NA	NA	NA	7
TanToCheung-2020	37	MIH/OH	24/96	20/81	63.0 (43–76)	62.0 (36–85)	4.5 (2.5–9.5)	4.8 (1–10)	20	88	18 (6)	75 (21)	NA	24	96	8
TruantS-2011	38	MIH/OH	36/53	31/47	60.6 ± 10.2	63.3 ± 7.6	2.9 ± 1.2	3.1 ± 1.2	3	4	34 (2)	44(9)	7 (19.4%)	36	53	9
TsutomuIwata-2018	39	MIH/OH	30/30	18/21	70 (19-86)	69 ((28-82)	2.4 (0.9-7)	2.4 (1.3-4.8)	7	6	29 (1)	28 (2)	NA	18	16	8
WeiLi-2018	40	MIH/OH	41/307	32/268	53.2 ± 11.1	54.3 ± 12.1	4.0 ± 2.0	5.7 ± 3.0	31	261	35 (6)	223 (84)	NA	NA	NA	7
WethitDum-2019	41	MIH/OH	41/41	28/35	73 (71–79	73 (71–75)	4 (2.5–5.7)	4.1 (2.7–7.0)	16	13	32 (9)	36 (5)	5 (12.2)	19	24	9
WuX-2018	42	MIH/OH	86/86	72/74	53 (17–79)	52 (21–82)	3.5 (0.9-12.5)	3.5 (0.8–11.3)	77	79	64 (22)	58 (28)	8 (9.3%)	86	86	9
XavierUntereiner-2018	43	MIH/OH	33/33	26/27	68.3 (61.0–73.2)	65.3 (64.3–71.5)	3.0 (2.3–5.0)	3.0 (2.0-4.8)	NA	NA	NA	NA	2 (6.1%)	24	21	7
XuHW-2018	44	MIH/OH	32/32	28/28	53.5 (26.0–70.0)	52.0 (27.0–74.0)	4.0 (1.0–10.0)	6.2 (1.5–10.0)	18	15	29 (3)	29 (3)	NA	NA	NA	7
YoonYI-2017	45	MIH/OH	33/33	23/26	56.03 ± 7.02	57.33 ± 6.88	3.31 ± 1.65	2.96 ± 1.5	29	28	NA	NA	NA	33	33	7
Young-InYoon-2019	46	MIH/OH	217/434	170/337	56.41 ± 9.65	56.94 ± 9.16	2.83 ± 1.28	2.90 ± 1.31	185	372	NA	NA	NA	145	284	7
YufuPeng-2019	47	MIH/OH	33/33	28/29	55.0 (35.0–76.0)	56.0 (29.0–74.0)	2.9 (1.0–3.0)	3.0 (1.0–3.0)	26	30	NA	NA	1 (3.0%)	27	24	8
Zhi-chengDeng-2018	48	MIH/OH	157/157	62/60	60(31–85)	61 (26–85)	2.47 (0.8–10)	2.86 (1–18)	140	143	138 (19)	135 (21)	NA	89	92	8

NA, not available.

## Meta−analysis results

### Operative time

**Figure 2:**
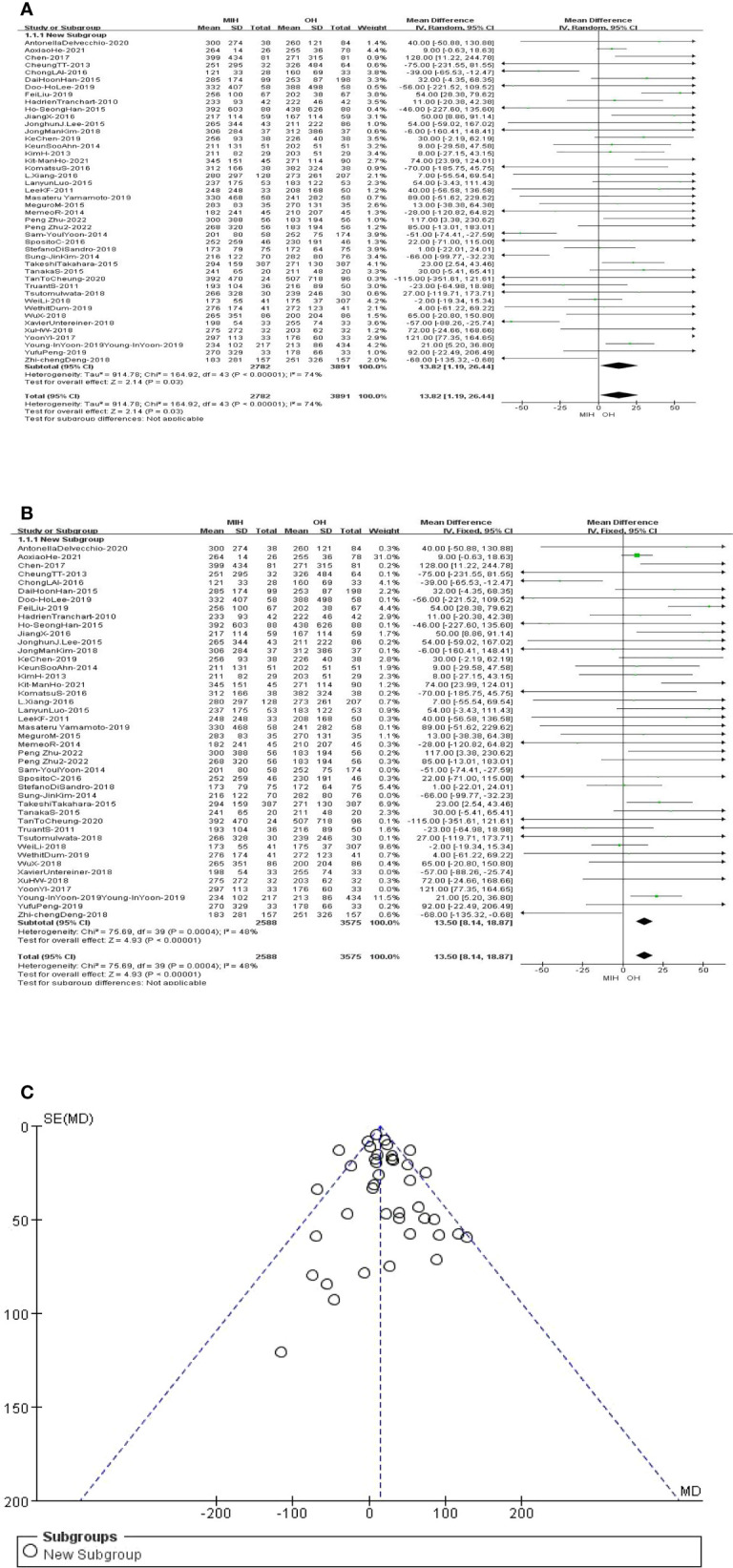
**(A)** Forest plot of operating time. **(B)** Forest plot of operating time after sensitivity analysis. **(C)** Funnel plot of operating time after sensitivity analysis.

All the studies (n = 6673 patients) compared the operation time in the meta-analysis. The analysis results showed that the operation time of OH group was slightly less than that of MIH group (Z=2.14, P=0.03, MD 13.82, 95% CI: 1.19–26.44). However, the data are highly heterogeneous (I^2^ = 74%, p<0.00001) (**Figure 2A**). Sensitivity analysis showed that the I^2^ value (I^2^ = 48%, p=0.0004) was less than 50 after removing 4 studies (Sam-2014, Sun-2014, Xavier-2018, YoonYI-2017) (31, 34, 43, 45), and the overall result was unchanged (**Figure 2B**). Funnel plot indicated there was no remarkable publication bias (**Figure 2C**).

### Blood loss

**Figure 3:**
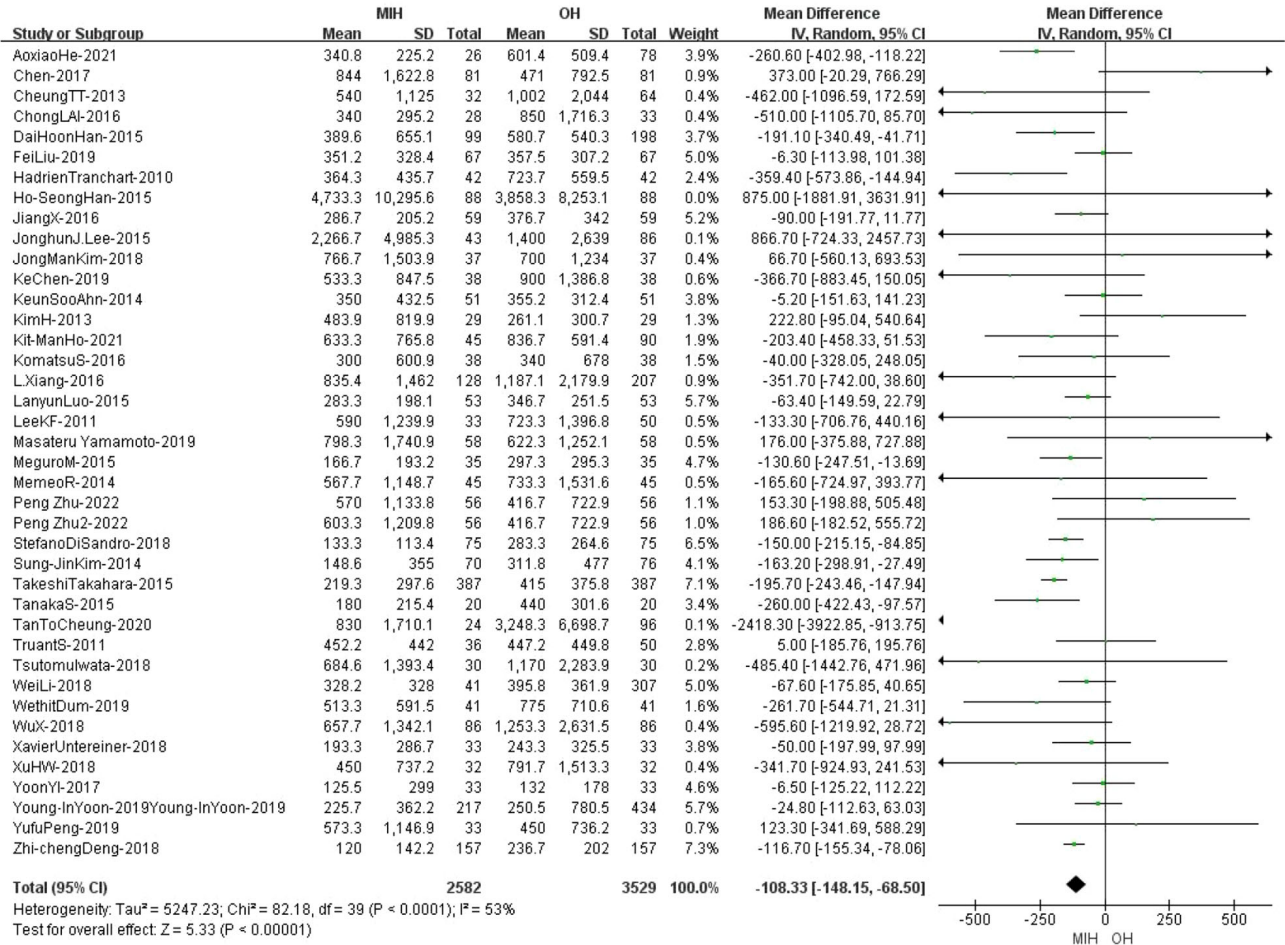
Forest plot of blood loss.

Blood loss was reported in 40 studies. The analysis results showed that the blood loss in MIH group was less than that in OH group (Z=5.33, P<0.00001, MD -108.33, 95% CI: -148.15 to -68.50). The data were highly heterogeneous (I^2^ = 53%, p<0.0001) (**Figure 3**). Sensitivity analysis showed that the I^2^ value (I^2^ = 46%, p=0.001) decreased when Takeshi’s study was removed (Takeshi-2015) (35). Then we applied the fixed effects model to the remaining studies and found that the final results did not change (**Supplementary Figure 1**).

### Length of hospital stay

**Figure 4:**
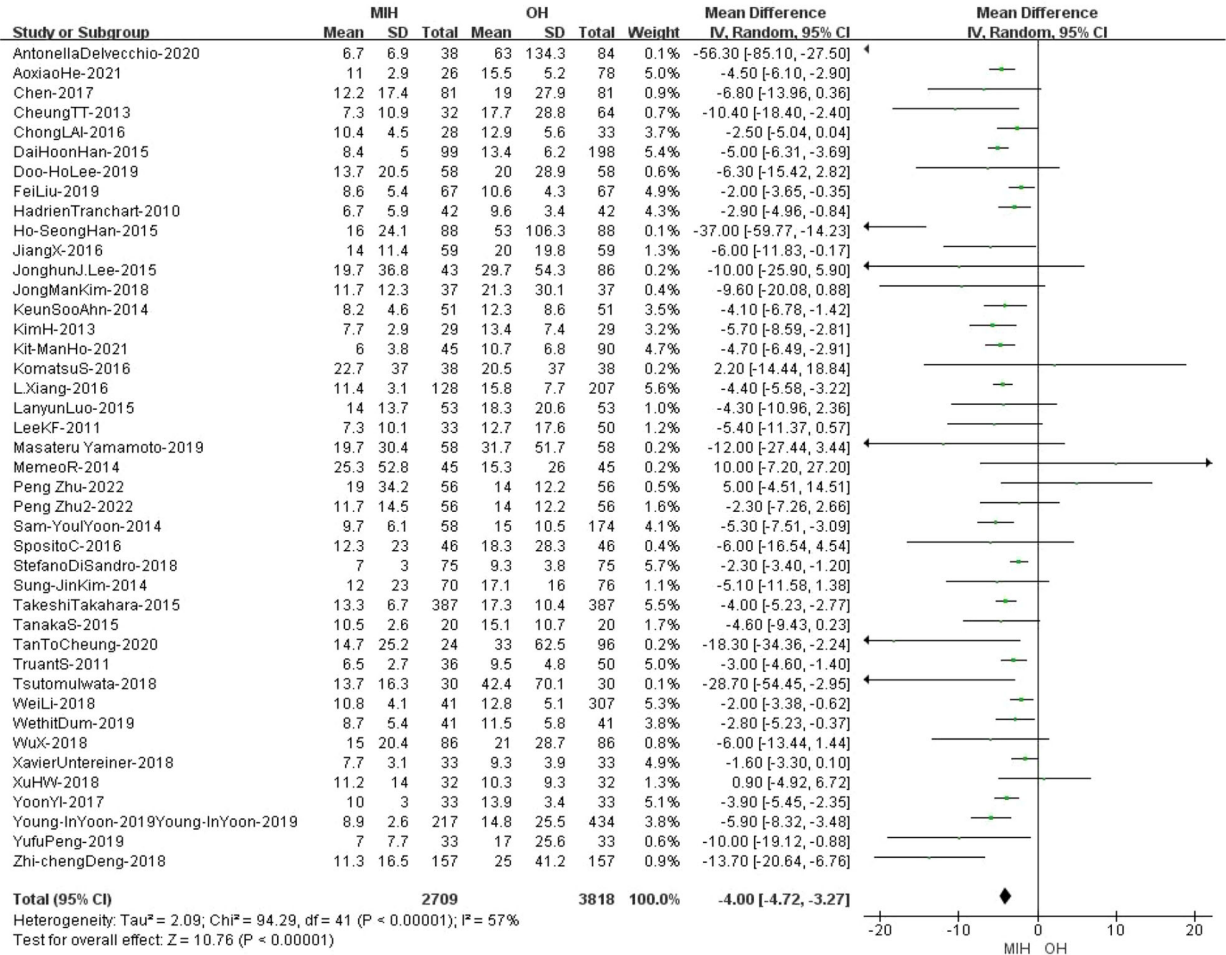
Forest plot of length of hospital stay.

42 studies (n = 6527 patients) compared the length of hospital stay in the meta-analysis. The analysis results showed that the length of hospital stay was shorter in the MIH group than in the OH group (Z=10.76, p<0.00001, MD -4.0, 95% CI: -4.72 to -3.27). Furthermore, the results indicated high heterogeneity (I^2^ = 57%, p<0.0001) (**Figure 4**). Sensitivity analysis showed that the I^2^ value (I^2^ = 37%, p=0.01) decreased when Antonella’s study was removed (Antonella-2020, Ho-SeongHan-2015, StefanoDiSandro-2018, Zhi-chengDeng-2018) (**Supplementary Figure 2**) (8, 16, 33, 48).

### Major hepatectomy

**Figure 5:**
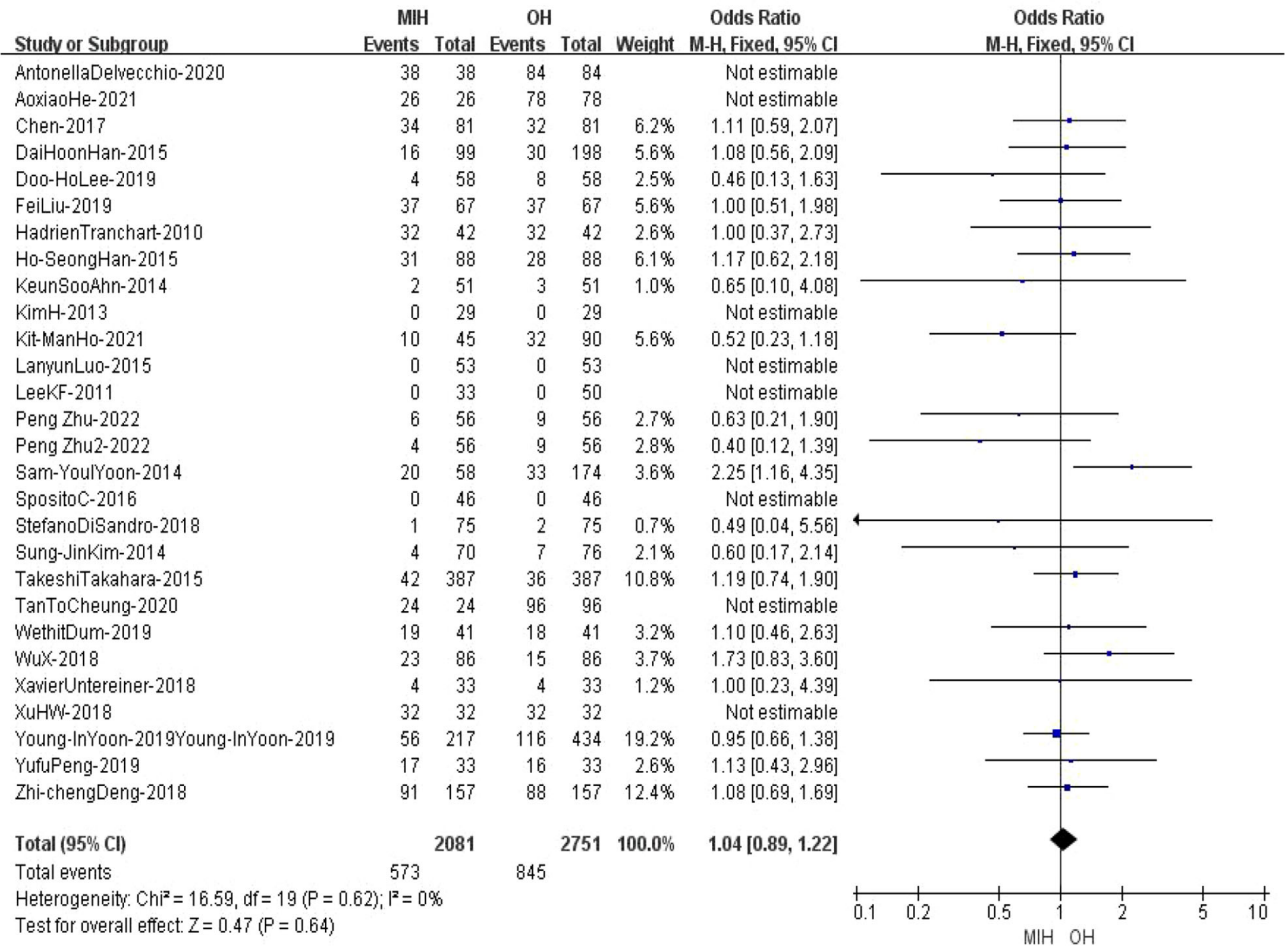
Forest plot of major hepatectomy.

28 studies (n = 4832 patients) compared major hepatectomy in the meta-analysis. The results of the analysis showed no significant difference between the two groups in major hepatectomy, with cases of 573 (2081) in the MIH group and 845(2751) in the OH group (Z=0.47, p=0.64, OR = 1.04, 95% CI 0.89 to 1.22, I^2^ = 0%) (**Figure 5**).

### Blood transfusion

**Figure 6:**
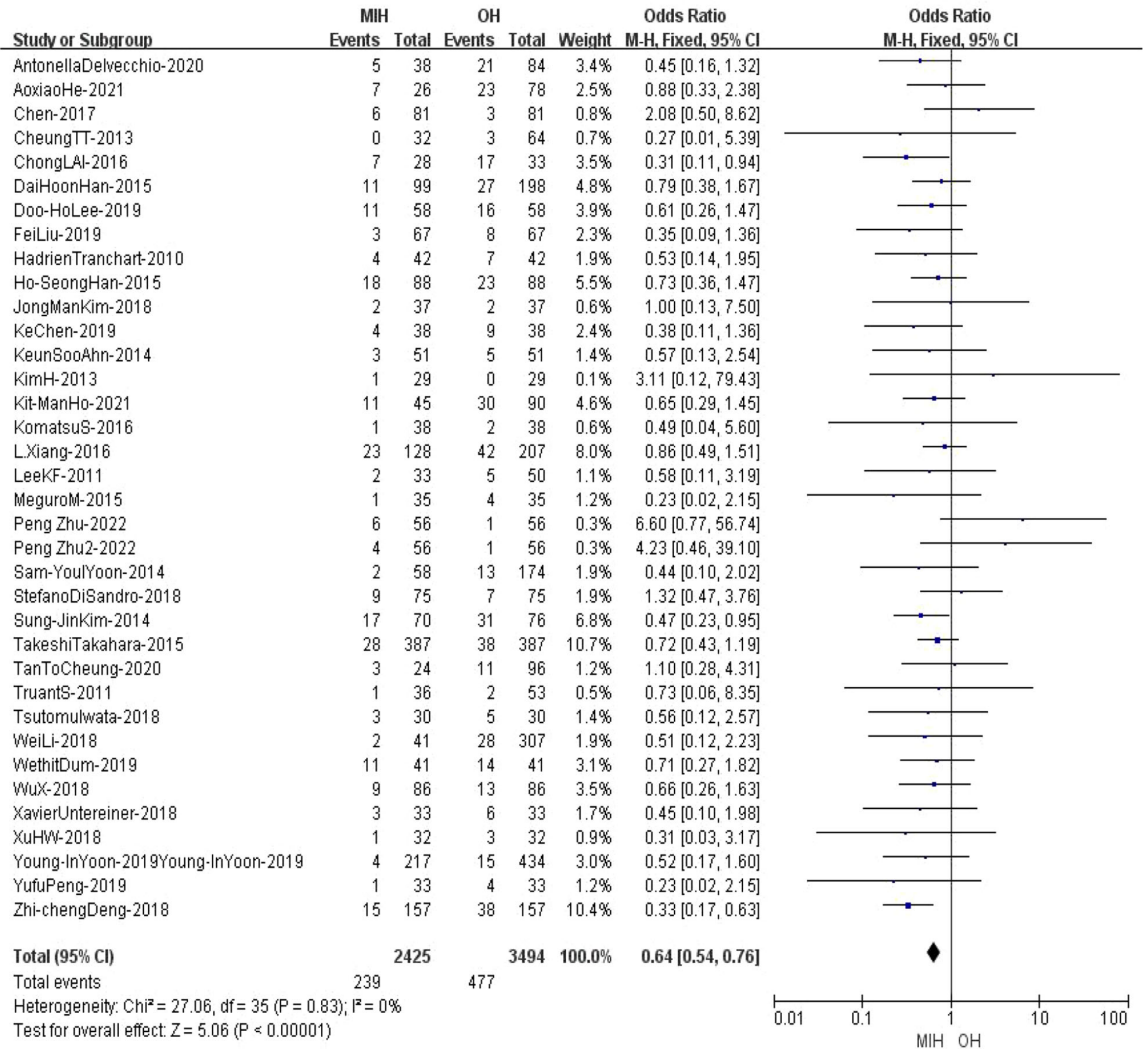
Forest plot of blood transfusion.

36 studies (n = 5919 patients) compared blood transfusion in the meta-analysis. The results of the analysis showed that the transfusion rate in (9.9%) MIH group was significantly lower than that in (13.7%) OH group (Z=5.06, p<0.00001, OR = 0.64, 95% CI 0.54 to 0.76, I^2^ = 0%) (**Figure 6**).

### Anatomical resection

**Figure 7:**
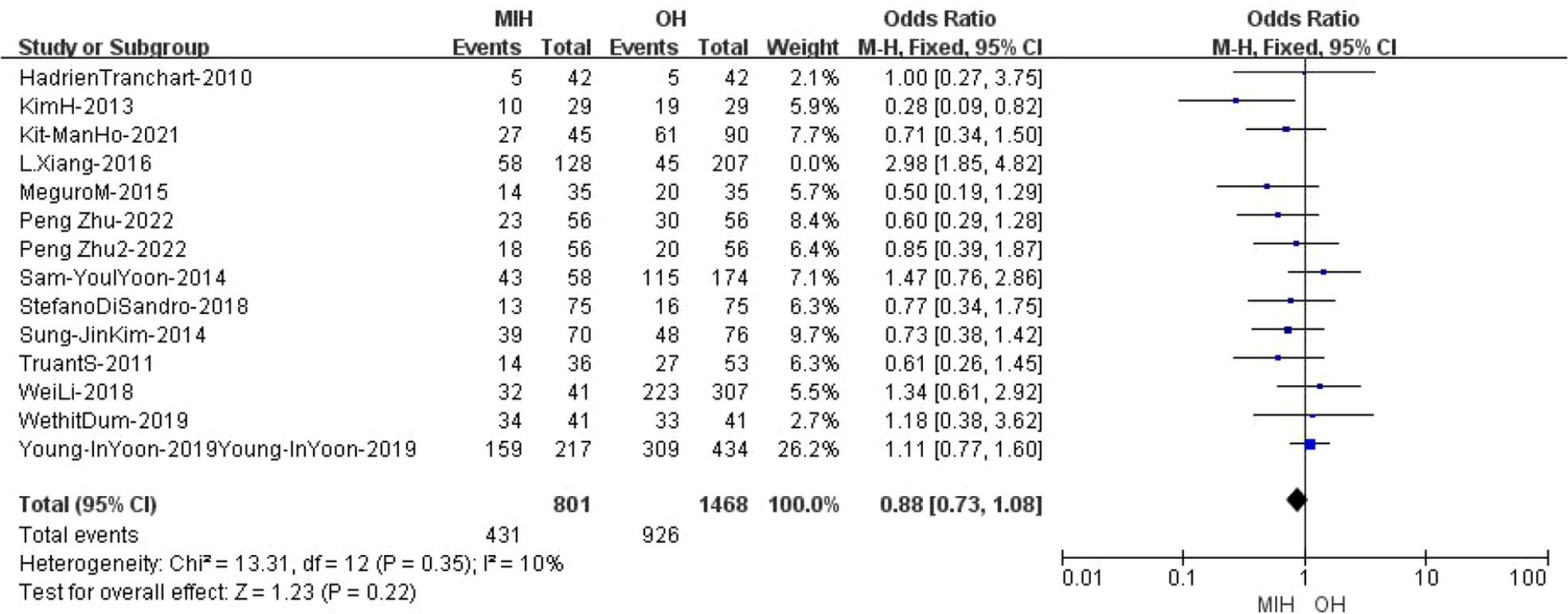
Forest plot of anatomical resection.

14 studies (n = 2604 patients) compared anatomical resection in the meta-analysis. The results of the analysis showed no significant difference between the two groups, with cases of 489(929) in the MIH group and 971(1675) in the OH group (Z=0.48, p=0.63, OR = 0.92, 95% CI 0.67 to 1.27). Furthermore, the results indicated high heterogeneity (I^2^ = 62%, p=0.001) (**Figure 7**). Sensitivity analysis showed that the I^2^ value (I^2^ = 10%, p=0.35) decreased when L.xiang’s study was removed (L.xiang -2016) (**Supplementary Figure 3**) (25).

### Satellite nodules

**Figure 8:**
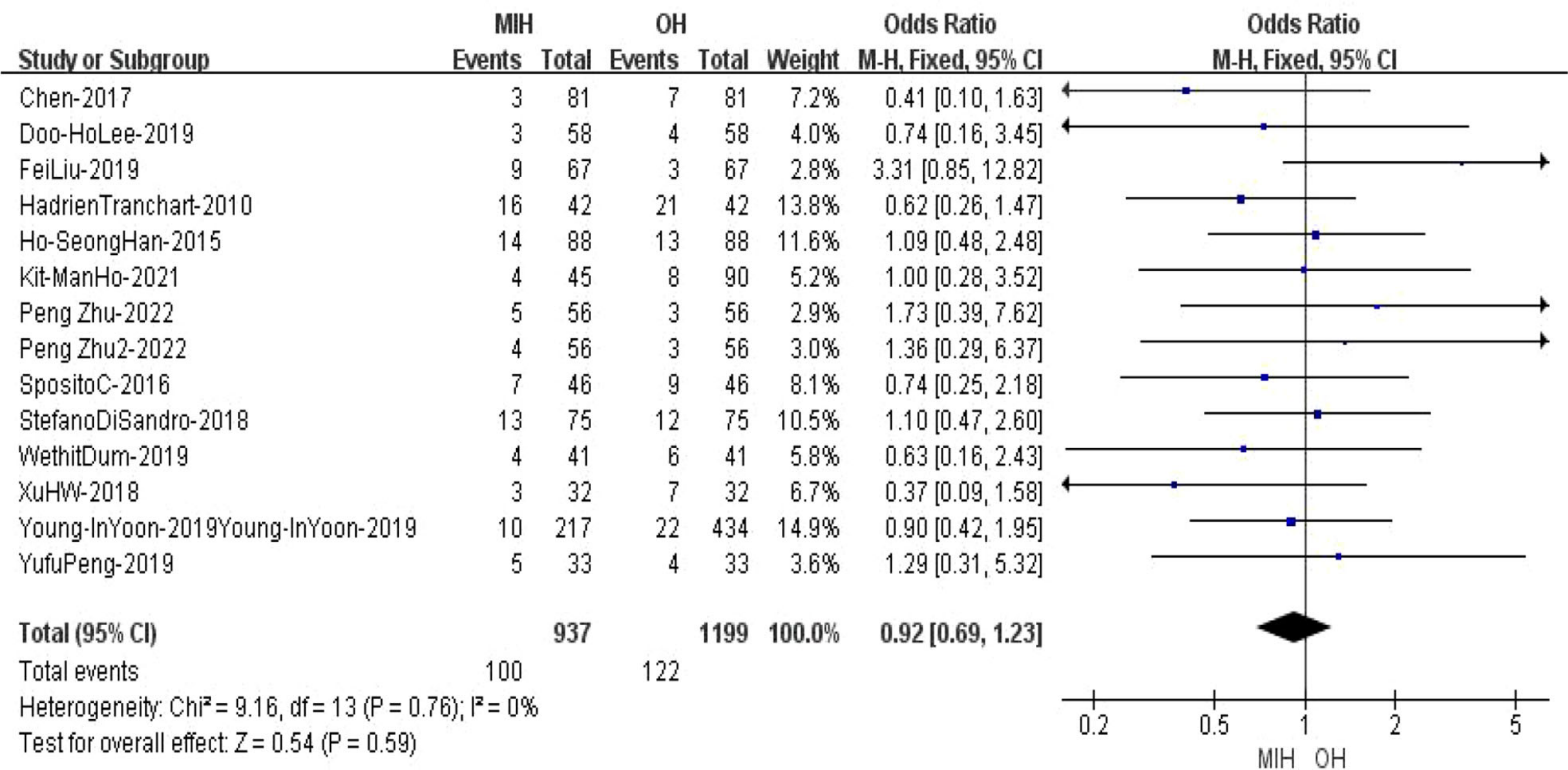
Forest plot of satellite nodules.

14 studies (n = 2136 patients) compared satellite nodules in the meta-analysis. The results of the analysis showed no significant difference between the two groups, with cases of 100(937) in the MIH group and 122(1199) in the OH group (Z=0.54, p=0.59, OR = 0.92, 95% CI 0.69 to 1.23, I^2^ = 0%) (**Figure 8**).

### Resection R0

**Figure 9:**
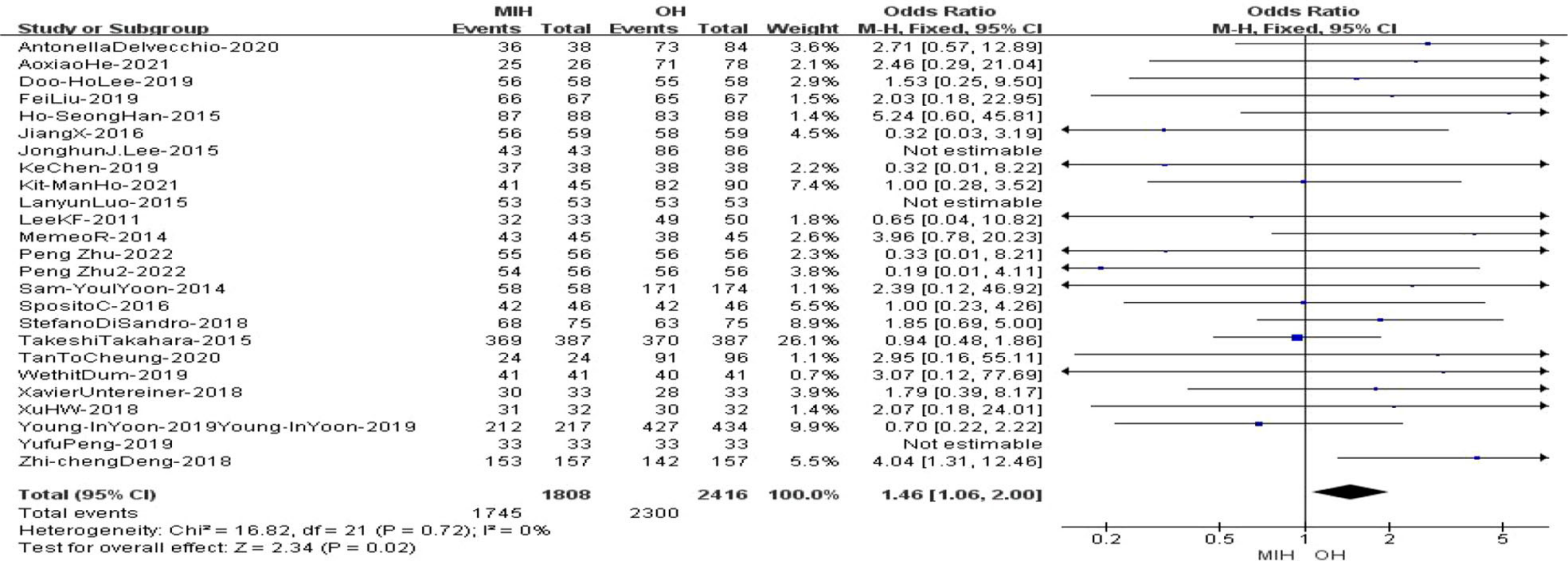
Forest plot of Resection (R0).

25 studies (n = 4224 patients) compared resection(R0) in the meta-analysis. The results of the analysis showed that the R0 rate in (96.2%) MIH group was higher than that in (94.8%) OH group (Z=2.34, p=0.02, OR = 1.46, 95% CI 1.06 to 2.0, I^2^ = 0%) (**Figure 9**).

### Microvascular invasion

**Figure 10:**
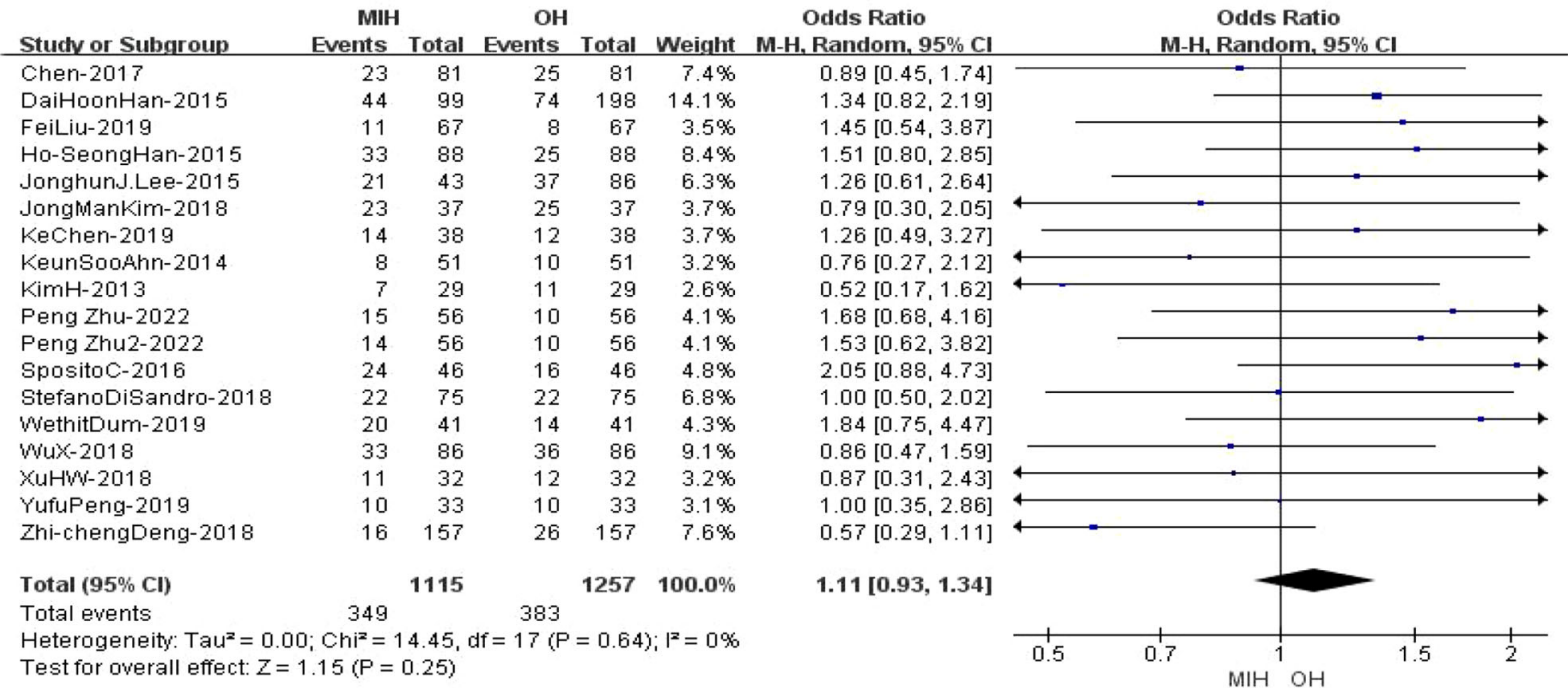
Forest plot of Microvascular invasion.

18 studies (n = 2372 patients) compared microvascular invasion in the meta-analysis. The results of the analysis showed no significant difference between the two groups, with cases of 349(1115) in the MIH group and 383(1257) in the OH group (Z=1.15, p=0.25, OR =1.11, 95% CI 0.93 to 1.34, I^2^ = 0%) (**Figure 10**).

### Postoperative complication

**Figure 11:**
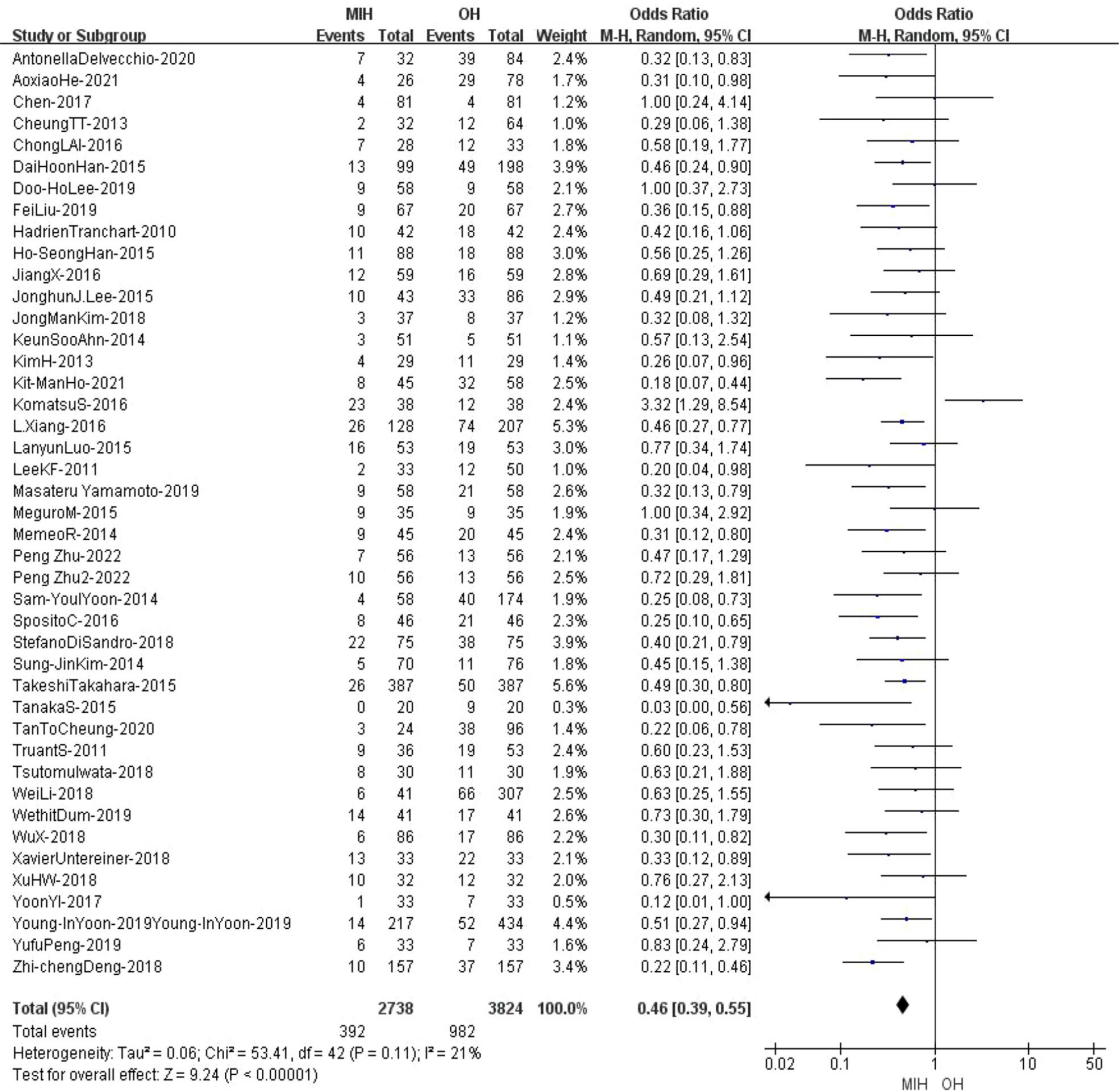
Forest plot of Postoperative complication.

43 studies (n = 6562 patients) compared postoperative complication in the meta-analysis. The results of the analysis showed that the overall morbidity in (14.3%) MIH group was significantly better than that in (25.7%) OH group (Z=9.24, p<0.00001, OR = 0.46, 95% CI 0.39 to 0.55, I^2^ = 21%) (**Figure 11**).

### Major morbidity

**Figure 12:**
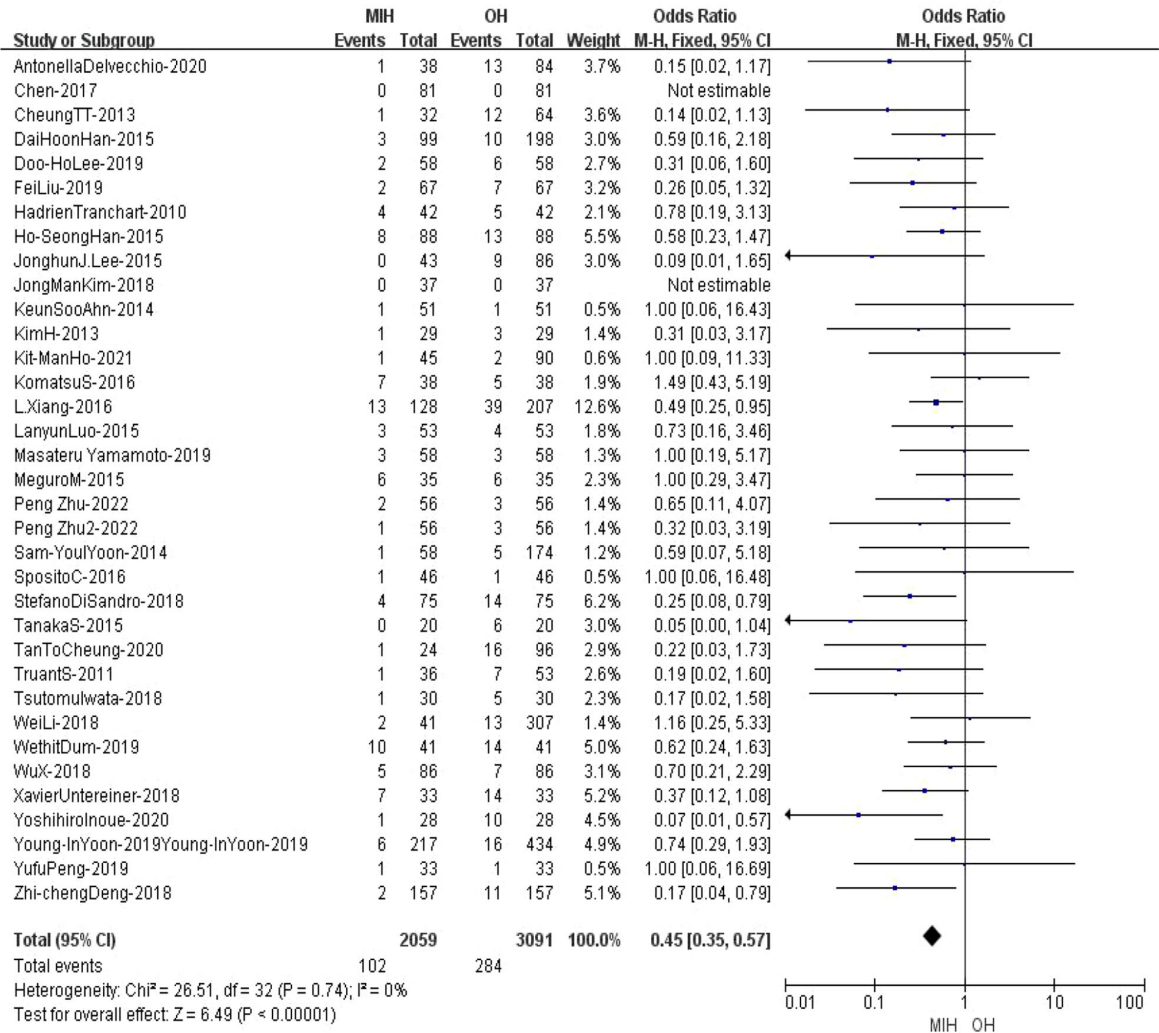
Forest plot of Major morbidity.

34 studies (n = 5094 patients) compared major morbidity(ClavienIII–IV) in the meta-analysis. The results of the analysis showed that the major morbidity in (5.3%) MIH group was significantly better than that in (9.6%) OH group (Z=6.11, p<0.00001, OR = 0.46, 95% CI 0.39 to 0.59, I^2^ = 0%) (**Figure 12**).

### Recurrence

18 studies (n = 2178 patients) compared recurrence in the meta-analysis. The results of the analysis showed no significant difference between the two groups, with cases of 364(936) in the MIH group and 483(1242) in the OH group (Z=0.71, p=0.48, OR =0.94, 95% CI 0.78 to 1.12, I^2^ = 19%) (**Supplementary Figure 4**).

### Overall survival

**Figure 13:**
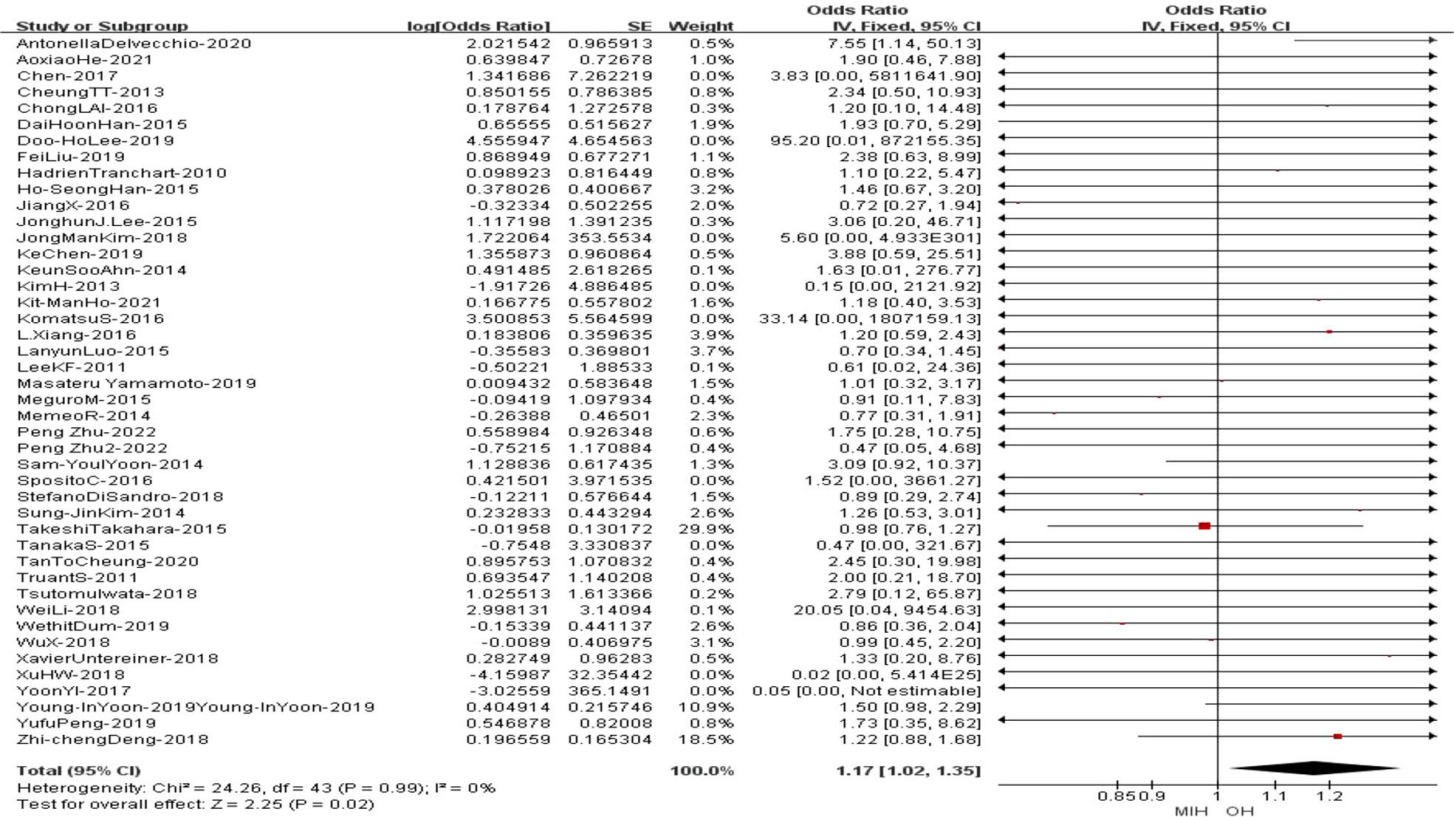
Forest plot of Overall survival.

All the studies reported the overall survival in the meta-analysis. The results of the analysis showed that the OS of MIH group was inferior to OH group (Z=2.25, p=0.02, HR = 1.17, 95% CI 1.02 to 1.35, I^2^ = 0%) (**Figure 13**).

### Disease free survival

**Figure 14:**
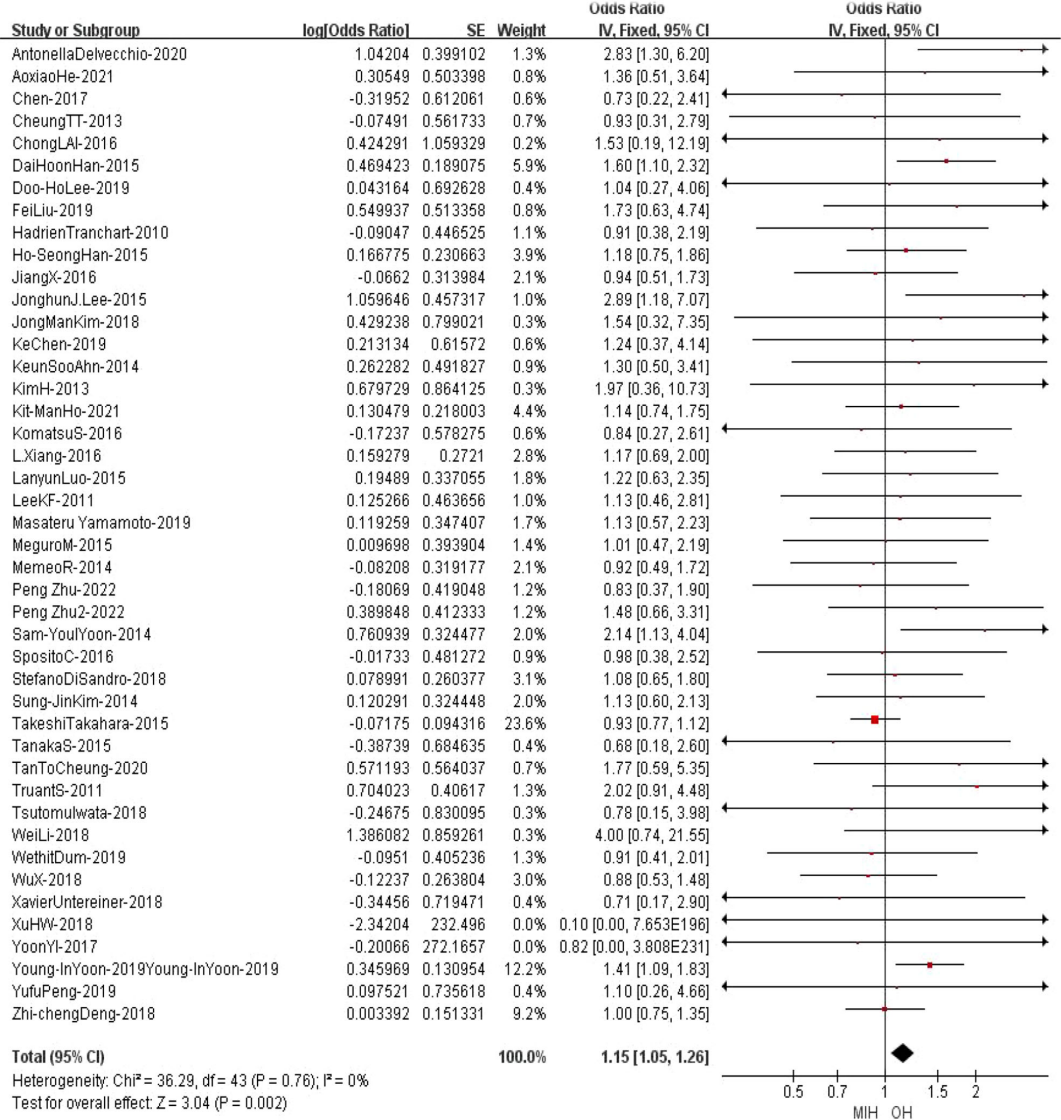
Forest plot of Disease-free survival.

All the studies reported the disease-free survival in the meta-analysis. The results of the analysis showed that the DFS of MIH group was inferior also to OH group (Z=3.04, p=0.002, HR = 1.15, 95% CI 1.05 to 1.26, I^2^ = 0%) (**Figure 14**).

## Discussion

The results of this systematic review and meta-analysis clearly illustrate the long-term survival outcomes of HCC treated with MIH and OH, which have been ambiguous for a long time. By integrating 43 high- quality case-control studies after propensity score matching, this paper found that traditional OH method had certain advantages over MIH in terms of long-term survival outcomes (OS and DFS). Although the gap between the two groups was not very obvious, it was still statistically significant. In the aspect of surgery, the results found that MIH has longer operation time, less blood loss and lower blood transfusion rate. In addition, there was no significant difference in major hepatectomy rate, anatomical resection rate between the two groups. In oncology, the results showed that there was no significant difference in satellite nodules rate and microvascular invasion rate between the two groups, but the R0 rate in MIH group was higher than that in OH group, and recurrence rate was lower than that in OH group. In terms of short-term postoperative results, the results showed that the length of hospital stay, postoperative complication rate and major morbidity rate in MIH group were lower than those in OH group.

The research results obtained from this meta-analysis verify the view put forward by most clinicians -MIH was a more challenging, complicated and delicate surgical operation, and it also reflects from the side that MIH can complete the surgical operation which is quite difficult as OH. And under the condition of the same oncology results, MIH has a better short-term postoperative effect. With the continuous progress of the times, surgical methods and instruments are also constantly evolving and developing, but the traditional hepatectomy still plays an irreplaceable role in some aspects at this present. At the same time, the narrow difference in survival outcomes between the two groups also proves that MIH has achieved good results after decades of development. However, at present, MIH technology has not fully reached the height of OH, and needs further improvement or change. For example, in some difficult operations, although MIH can complete the whole operation, the operation time is long; the intraoperative visual field is not as good as OH, and some complex situations cannot be seen; the operation space in the body is limited, and the complex surgical process cannot be completed; all these will affect the patient’s OS and DFS. In addition, open hepatectomy can touch organs more intuitively. If there is more local bleeding during the operation, we can use hand compression to stop bleeding quickly and effectively, and local adhesion can also be touched by hand to separate adhesion in time, so as to avoid other unnecessary injuries. For now, traditional OH has certain advantage in survival outcomes, but we do not know the future results. We will continue to pay attention to and study whether this advantage will continue. In this study, we found that the R0 rate of MIH is higher than that of OH, and it is statistically significant. This may be due to the continuous improvement of other auxiliary surgical equipment, such as the widespread application of intraoperative ultrasound technology in major centers. In addition, although MIH sacrifices a wide surgical field of vision, enlarged intraoperative vision and clear intraoperative images can promote surgeons to perform surgical operations more accurately, which is conducive to the resection of tumor tissue; However, it must be stated that the literatures included in this study are not RCT studies, and there may be bias in the selection of patients, which may affect the R0 results.

From the first case of OH in 1886 to the successful implementation of LH in 1991 to the first reported RD in 2006, human beings have created miracles again and again, amazing the world (50). As the most primitive procedure of hepatectomy, OH technology has been skillfully mastered by surgeons and widely used in clinical practice. However, its shortcomings such as high intraoperative blood loss, large postoperative trauma and high incidence of complications are becoming more and more obvious, and permanent large incision scars will undoubtedly bring physical and mental pressure to young women (51). As we all know, since the 21st century, “precise and minimally invasive surgery” has become a trend in the field of surgery. In 2008, the first international consensus conference of laparoscopic hepatectomy put forward the basic indications of LH: the single lesion range ≤5cm, and the lesion is mainly located around the liver (2-6 segments) (52). In 2014, the second international consensus conference of abdominal hepatectomy proposed that laparoscopic small-scale hepatectomy can become a standard operation, and laparoscopic large-scale hepatectomy needs further exploration (53). Nowadays, more and more patients are interested in MIH technology, and MIH has been unanimously recognized and widely accepted. Compared with OH, LH has a flexible and clear vision, which makes it possible to dissect blood vessels, bile duct structures and ligaments around the liver in detail, thus reducing intraoperative bleeding and postoperative complications such as bile leakage, ascites and bleeding (54). Robot technology began to be used in general surgery in the 1990s. Since its establishment, DaVSS has been widely used in a variety of clinical diseases, including gastrointestinal tract, hepatobiliary pancreas, genitourinary and other disciplines. DaVSS can provide surgeons with 10-15 times of three-dimensional and clear surgical vision (55). Moreover, the flexible robotic arms can “ fight left and right”, and the seven degrees of freedom can break the limit of manual wrist rotation operation, so as to complete the delicate operation in the narrow anatomical area. However, a series of problems, such as the defect of touch temperature feedback system, the inconsistency between surgical instruments and surgical methods, and the standardization of surgical techniques, need to be further explored and improved by clinicians. At present, there are few literature reports on the application of RH in HCC, and RCT studies are even less. It is believed that major centers are in the period of summarizing experience, and more high-quality studies on RH in the treatment of HCC are expected to be reported.

As far as we know, this study is the first to compare the clinical efficacy of MIH and OH in the treatment of HCC based on high-quality propensity score matching studies. However, there are still some limitations in this study. First of all, the included literature is observational case-control studies and lacks substantial evidence from randomized controlled trials, which may lead to the occurrence of patient selection bias. Secondly, some data need to be converted by the formulas of Wan X and Tierney JF to meet the input requirements, which may cause errors, but we choose the conversion formulas recommended by PRISMA. Third, there are few reports about the application of RH in HCC, and most of the data included in this study are LH, so there is no further subgroup analysis of MIH, and it is not clear whether there is a difference between RH and LH. Finally, most of the studies included in this paper are post-2010 articles, which may produce some impacts on the results, but most of the previous literature contain mixed data that do not meet the inclusion criteria and are excluded because of low quality.

Through the efforts of several generations of hepatobiliary surgeons, MIH technology has been widely used in related liver diseases, and its feasibility, safety and efficacy have significant advantages. Of course, OH, as the original basic surgical method, still plays an irreplaceable role in some specific situations.

## Data availability statement

The original contributions presented in the study are included in the article/**Supplementary Material**. Further inquiries can be directed to the corresponding author.

## Author contributions

BF: literature review, statistical analysis, drafting of manuscript. P-SH: literature review, data extraction. J-RZ: literature review, adjudication, data extraction. Y-MZ: concept and design, critical revision of manuscript. All authors contributed to the article and approved the submitted version.

## Funding

This work is supported by the Tianjin Health Science and technology project, No. TJWJ2021ZD002; Science and Technology Planning Projects of Tianjin, No. 19ZXDBSY00010; Science and Technology Project of Tianjin Health Commission, No. ZC20174; Tianjin Natural Science Foundation, No. 20JCYBJC01310; Tianjin Health Science and technology project, No. ZC20218; Tianjin Health Science and technology project, No. ZC20064.

## Conflict of interest

The authors declare that the research was conducted in the absence of any commercial or financial relationships that could be construed as a potential conflict of interest.

## Publisher’s note

All claims expressed in this article are solely those of the authors and do not necessarily represent those of their affiliated organizations, or those of the publisher, the editors and the reviewers. Any product that may be evaluated in this article, or claim that may be made by its manufacturer, is not guaranteed or endorsed by the publisher.
