# Supplementary figures and images for: Comparison of survival and post-operation outcomes for minimally invasive versus open hepatectomy in hepatocellular carcinoma: A systematic review and meta-analysis of case-matched studies
Source: Front Oncol. 2022 Oct 20;12:1021804. doi: 10.3389/fonc.2022.1021804 (PMC9633112; doi:10.3389/fonc.2022.1021804)

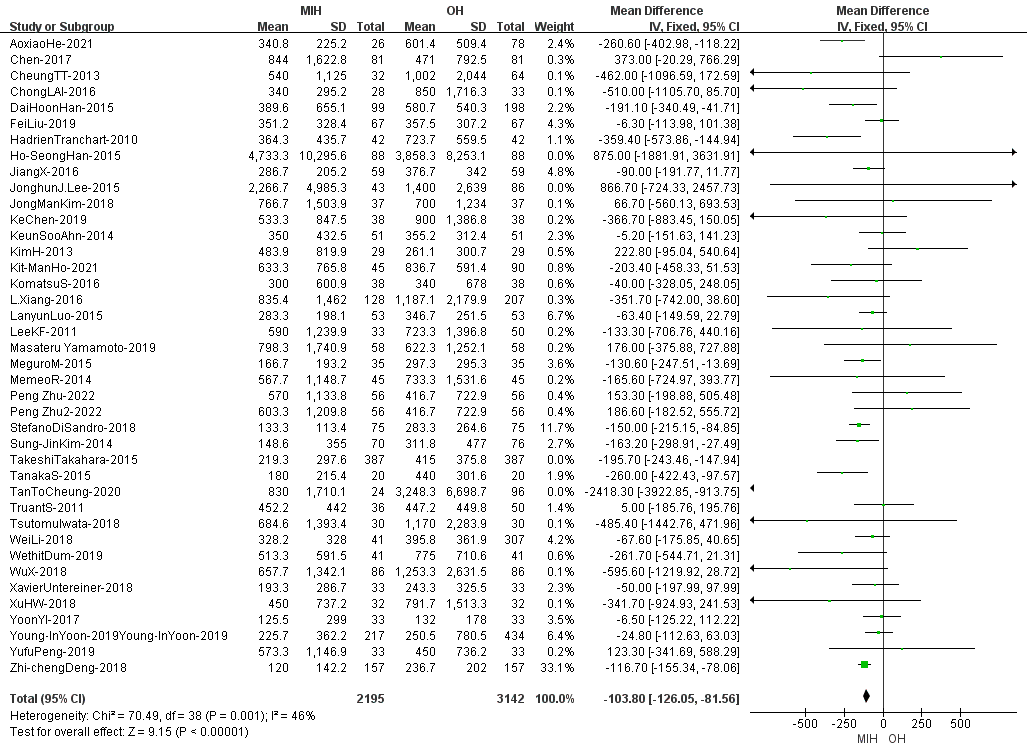

Supplement: Supplementary file 2 [file Image_1.jpeg]

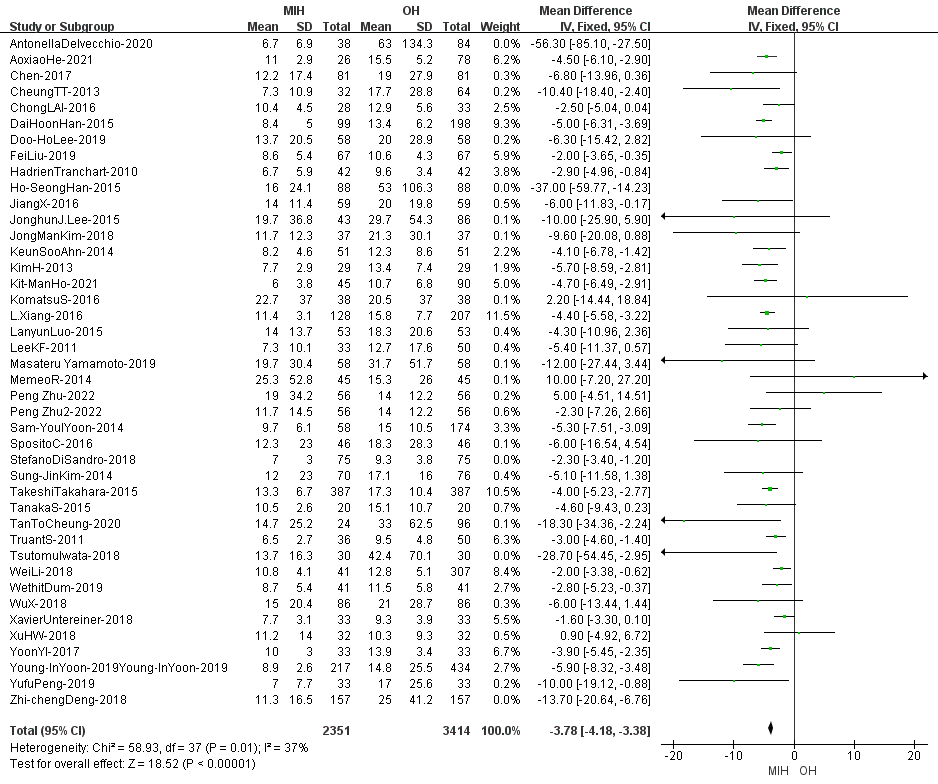

Supplement: Supplementary file 3 [file Image_2.jpeg]

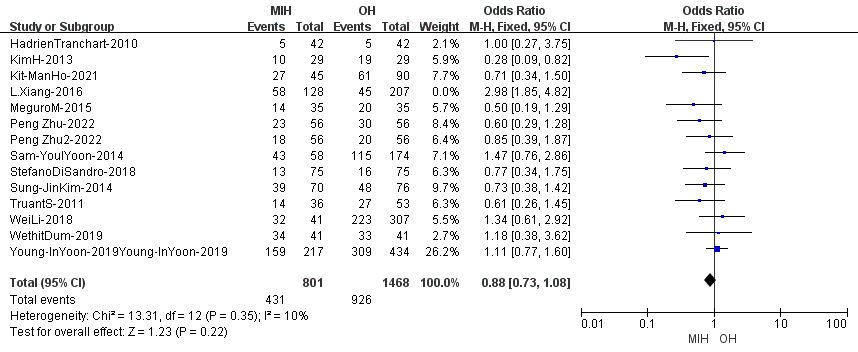

Supplement: Supplementary file 4 [file Image_3.jpeg]

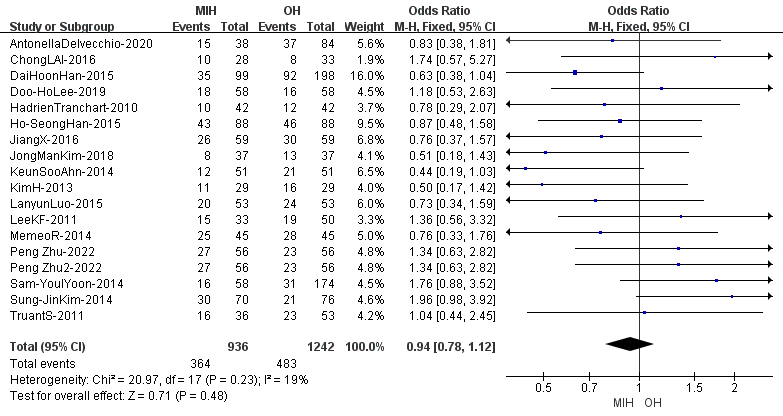

Supplement: Supplementary file 5 [file Image_4.jpeg]
